# Metagenomic Data Assembly – The Way of Decoding Unknown Microorganisms

**DOI:** 10.3389/fmicb.2021.613791

**Published:** 2021-03-23

**Authors:** Alla L. Lapidus, Anton I. Korobeynikov

**Affiliations:** Center for Algorithmic Biotechnology, St. Petersburg State University, Saint Petersburg, Russia

**Keywords:** metagenomics, metagenomic assembly, microbiota, algorithms, SPAdes

## Abstract

Metagenomics is a segment of conventional microbial genomics dedicated to the sequencing and analysis of combined genomic DNA of entire environmental samples. The most critical step of the metagenomic data analysis is the reconstruction of individual genes and genomes of the microorganisms in the communities using metagenomic assemblers – computational programs that put together small fragments of sequenced DNA generated by sequencing instruments. Here, we describe the challenges of metagenomic assembly, a wide spectrum of applications in which metagenomic assemblies were used to better understand the ecology and evolution of microbial ecosystems, and present one of the most efficient microbial assemblers, SPAdes that was upgraded to become applicable for metagenomics.

## Introduction

The increased scientific and practical interest in the microbial world that surrounds us, as well as the emergence of new molecular-biological and bioinformatic approaches for the analysis of the diversity and genetic potential of microbial communities from diverse environments gave rise to what is now known as metagenomics.

While partial sequence of microbiotal DNA (community of microorganisms) is sufficient to assess the information about the diversity of the sampled community, to uncover the genetic potential, we need to analyze the extended genomic regions, or even better, fully restored genomes from the microbiome (combined genome of the microbiota). These types of extended regions can be obtained by assembling short DNA fragments produced by modern sequencing technologies.

Assembling a genome is a difficult task both due to the complexity of the specific genomes and the many particularities of the sequencing technologies used to accomplish this goal. In the case of metagenomic data, this task is further complicated by (1) the large volume of data produced; (2) the quality of the sequence; (3) the unequal representation of members of the microbial community; (4) the presence of closely related microorganisms with similar genomes; (5) the presence of several strains of the same microorganism; and (6) an insufficient amount of data for minor community members (Kunin et al., 2008; Sczyrba et al., 2017).

To overcome (or at least partially solve) these problems, a number of various approaches and analytical pipelines have been created and applied, with new ones being continuously worked on (Ayling et al., 2020).

Here, we describe the challenges of metagenomic assembly (MA), the role MA played in the revolutionary discoveries of recent years, expanding our knowledge of the microbial world of our planet.

## Main Approaches

There are two main approaches to the analysis of natural microbial communities: amplicon sequencing (mainly of 16S rRNA or 18S rRNA) and random shotgun metagenomic sequencing of the aggregated metagenomic DNA (mgDNA) of the entire microbial community (Breitwieser et al., 2019).

The amplicon-based approach combines sequences based on their similarity to reference sequences (such as 16S rRNA gene sequence) or to operational taxonomic units (OTUs; Schloss and Westcott, 2011). Its success heavily depends on the sequencing platform being used and the level of completeness of the reference database (3). An incomplete reference database makes the task of analyzing novel sequences belonging to previously unidentified taxonomic lineages virtually impossible (Schloss and Westcott, 2011; Tyler et al., 2014).

The 16S rRNA analysis is fairly fast and cheap and provides reliable information on the composition and diversity of the community, but is not able to analyze the biological functions neither of the individual community members, nor of the community as a whole. This approach can also not be used for detecting extra chromosomal elements of a genome (plasmids) or identify viruses. Moreover, it was also demonstrated that the universal PCR primers traditionally used for PCR amplification of the segments of the 16S rRNA genes (mostly V1–V3, V4, and V4–V5 regions) are not actually universal and thus fail to fully represent the community’s composition (Fuks еt al., 2018).

Shotgun metagenomic sequencing allows to overcome some of the limitations of the 16S rRNA analysis. Metagenomic analysis of microbial communities answers three questions: “who is there?” by performing taxonomic characterization of the community, “what do they do?” by providing functional annotation using contigs from metagenomic assemblies, and “how do they differ?” by comparing them via comparative analysis. On top of that metagenomic data allow to identify strains of common species by gene content, which is not possible with the 16S rRNA sequencing approach.

For a thorough analysis of the community, metagenomic shotgun reads can be assembled with the help of available reference genomes (reference-based assembly) or *de novo* (*de-novo* assembly).

There are many genome assemblers specifically designed for metagenomic data (Vollmers et al., 2017), but none of them are perfect. The development of metagenomic assemblers is a challenging and important task that depends on the complexity and diversity of microbial communities, the abundance of community members, and the amount and quality of available experimental data.

A typical metagenomic project consists of the following steps:

- sample and metadata collection
- DNA extraction
- community composition analysis using 16S rRNA gene
- whole metagenome sequencing library construction
- sequencing
- read preprocessing
- assembly
- gene-calling on reads, contigs, or both
- binning (depending on the approach, this step can be applied prior to and/or after assembly)

The success of the bioinformatic analysis heavily depends on the proper planning of the experiments and implementation of the metagenomic projects.

## Challenges of Metagenome Assemblies

Ideally, the result of a metagenome assembly process should be a set of genomes of all the species that are represented within the input data. In reality, this end result is still very difficult to achieve, since this approach is still very new and for the moment works best for ordinary bacterial isolate data without manual curation and only with a very specific combination of input data at that. To explore the subject matter further, we will split all the challenges of metagenome assemblies into three separate groups, namely:

1. challenges of the assembly process
2. challenges of generating assembly results
3. challenges of result usage

### Challenges of the Assembly Process

There are many properties of input data that significantly complicate the metagenomic assembly process itself making it much harder than conventional bacterial isolate assemblies.

Firstly, the widely different abundance levels of various species in a metagenomic sample result in a highly non-uniform read coverage across different genomes. Moreover, coverage of most species in a typical metagenomic dataset is much lower than in a typical sequencing project of a cultivated sample. As a result, standard assembly techniques aimed at isolate genomes with high and rather even coverage, tend to generate fragmented and error-prone metagenomic assemblies.

Secondly, one of the main challenges of the assembly of single species is the correct placement of repetitive DNA segments within a genome (*repeat resolution* process). For a single organism, assuming a uniform read coverage, such repeats can be detected using the elevated read coverage depth, i.e., a repeat having a multiplicity of two (so, two copies in a genome) would be covered by twice as many reads as expected, simply because we cannot distinguish between different copies of the repeat. Due to the uneven (and unknown) representation of the different species within a metagenomic mixture, a simple coverage-based approach can no longer be used to detect the repeats. Furthermore, various species within a microbial community often share highly conserved genomic regions, representing, for example, mobile elements or the genes shared due to horizontal gene transfer. Besides complicating the assembly and fragmenting contigs, such “interspecies repeats,” together with low coverage of most species, may trigger intergenomic assembly errors.

Thirdly, metagenomic data consists of a mixture of DNA from different organisms, and may include viral, bacterial, or eukaryotic organisms. Recent applications of single-cell (Kashtan et al., 2014) technologies revealed an enormous microdiversity of related strains within various microbial communities. While strains share most of the genomic sequence, they often have significant variations arising from mutations, insertions of mobile elements, genome rearrangements or horizontal gene transfer. Overall, many bacterial species in a microbial sample are represented by strain mixtures, i.e., multiple related strains with varying abundances (Kashtan et al., 2014; Sharon et al., 2015). Although various studies outside the field of metagenomics extensively addressed a similar challenge of assembling two haplomes within a highly polymorphic eukaryotic genome (Donmez and Brudno, 2011; Kajitani et al., 2014), assembly of many closely related bacterial strains is a somewhat different problem with its own set of unique computational challenges.

Finally, the necessity to reconstruct even rare strains from a strain mixture often leads to an enormous size of input data. In such cases, some genomes could be sequenced to a very high coverage depth (often exceeding 1000x). Depending on the particular assembly algorithm used, such situations can easily lead to a very high computation cost (Li et al., 2012). For the overlap-layout-consensus assembly approach, elevated coverage depth leads to a quadratic increase in the time necessary to compute overlaps (and in the number of overlaps that need to be computed). For de Bruijn graph assemblers, the very high coverage depth amplifies the effect of errors on the assembly graph and may even confuse error correction algorithms (simply by chance multiple random errors can “confirm” each other; Rizzi et al., 2019). Also the level of complexity (the number of different species) of a metagenome could be very different, from tens to ten thousands even further complicating the assembly process.

### Challenges of Generating Assembly Results

As we already mentioned, the microbial communities often contain many closely related strains. Differences between these strains prevent assemblers from identifying a consistent path through the assembly graph, leading to potentially fragmented assemblies, or even worse, lead to misassemblies.

In particular, such differences often result in “bulges” and “tips” in the de Bruijn graphs that are very similar to the artifacts caused by sequencing errors (Zerbino and Birney, 2008). For example, sequencing errors often result in a bulge formed by two short alternative paths between the same vertices in the de Bruijn graph, forming a “correct” path with high coverage and an “erroneous” path with low coverage. Similarly, a substitution or a small indel in a rare strain (as compared to an abundant strain) often results in a bulge formed by a high-coverage path corresponding to the abundant strain and an alternative low-coverage path corresponding to the rare strain. As a result, metagenomic assemblers need to be very careful so as to not confuse sequencing errors with similar artifacts caused by the differences in low-abundant strains.

To partly address this problem metagenomic assemblers typically aim to achieve a so-called *consensus assembly* (though strain-oriented assemblers certainly do exist, see Gregor et al., 2016) by trying to detect and mask variations between closely related strains. Consensus assemblies as opposed to *strain assemblies* have both advantages and disadvantages for downstream analysis.

Strain assemblies:

- Pros: ability to accurately reconstruct information about individual strains
- Cons: assembly quality is often inferior due to long unresolved repeats

Consensus assemblies:

- Pros: ability to reconstruct the backbone structure of the species genome
- Cons: can result in annotation artifacts and lose information about individual strains

So, in order not to confound the results of downstream analysis, it is extremely important to understand what particular type of results is produced by the assembler. In addition to this, the performance of different assemblers could be very different: Olson et al. (2019) demonstrated that on (relatively simple) HMP metagenome, MEGAHIT has fewer structural errors compared with metaSPAdes, while metaSPAdes has fewer under-collapsed/over-collapsed repeats when compared with MEGAHIT. These differences might be important depending on the particular steps of downstream analysis.

### Challenges of Result Usage

Typically, the result of an assembly process is a set of contigs or scaffolds. We already saw that there might be quite a variation here, because these contigs could represent strain assemblies or consensus assemblies. However, contigs and/or scaffolds are not able to represent the result of assembly in its entirety since they cannot account for the important contiguity information: while metagenome assemblers certainly use read pairs to resolve possible repeats, usually the repeat structure of the metagenome is very complex as in addition to the within-species repeats an assembler have to deal with interspecies repeats caused by horizontal gene transfer, conservative regions, and closely-related strains. Metagenome assemblies are done from short read-pairs having the fragment length of less than 1,000 bp. As a result, the repeat resolution possibilities available to an assembler are quite low and many repeats are left unresolved. In this sense, oftentimes scaffolds are almost near identical to the contigs for metagenomic assemblies and the use of, e.g., external scaffolding tool is hopeless and could even lead to additional misassemblies due to all repeat ambiguities in the complex metagenomes.

Often in addition to a set of contigs, assemblers also produce assembly graphs and the necessary mapping between the contigs and paths through the assembly graph. The assembly graph and contig paths are usually provided in GFA format (Gonnella and Kurtz, 2016; Gonnella et al., 2019), a format that has quickly become the standard de-facto for representing assembly graphs. Subsequently, many different tools (such as, for example, Bandage; Wick et al., 2015) could be used to visualize the resulting graph.

Unfortunately, while there are many tools to manipulate and visualize assembly graphs, there are very few tools that could utilize them as inputs. Certainly, assembly graphs are much more complex objects to analyze as compared to ordinary contig sequences. However, as metagenomic assemblies are often very fragmented, the use of the additional contiguity information encoded in the graphs could be crucial for obtaining accurate results from downstream tools. Fortunately, several tools have recently emerged that could utilize the assembly graph as an input. We outline SPAligner – a tool for aligning the nucleotide and amino-acid sequences directly to the assembly graph (Dvorkina et al., 2020) and Pathracer – a tool for the alignment of profile Hidden Markov Models (HMMs; both nucleotide and amino-acid) to assembly graphs (Shlemov and Korobeynikov, 2019). GraphBin2 (Mallawaarachchi et al., 2020) uses the connectivity and coverage information from assembly graphs to adjust existing binning results on contigs and to infer contigs shared by multiple species, thus significantly increasing the completeness of the bins and corresponding metagenome-assembled genomes (MAGs).

Still, in many cases, there is a need to integrate downstream analysis tools somewhere in the middle of the assembler pipeline. This approach could benefit both steps: the tool could gain access to all of the information available to the assembler (e.g., assembly graph, coverage information, paired-end read alignments, etc.), while the assembler could reuse the information provided by the tool. Among the first examples of such instruments was plasmidSPAdes (Antipov et al., 2016a) – a tool for the extraction and assembly of putative plasmid components from an assembly graph. Recent examples of such tools include metaplasmidSPAdes (Antipov et al., 2019), capable of assembling putative plasmid sequences from metagenome assemblies, and biosyntheticSPAdes (Meleshko et al., 2019) that aims to reconstructing the backbone structure of secondary metabolite synthetases (e.g., NRPS/PKS) from metagenome assemblies.

## Ways to Overcome Metagenomic Assembly Challenges

A good number of specialized metagenomic assemblers were developed to solve the problems of metagenomic assembly caused by the features of the data being collected (Ayling et al., 2020). Depending on the length of the reads created, assemblers are based on different approaches – from overlap-layout-consensus tools based on overlap strategies (Laserson et al., 2011), to those that use de Bruijn graphs to work with data (Li et al., 2015b). Important to mention, that not only efficiency and quality of work affect assemblers’ popularity, but also the ease of use of the tool, the presence of a simple, detailed, easy to read manual, the ongoing development of the program and the speed and quality of the feedback from the tool support team. The combination of all these factors made MEGAHIT (Li et al., 2015b) and metaSPAdes (Nurk et al., 2017) the most used assemblers among other popular ones, such as IDBA-UD (Peng et al., 2012), Ray Meta (Boisvert et al., 2012), and others. During the past few years, many tools that could contribute to metagenomic assemblies have been developed (see Ayling et al., 2020 for more-than-exhaustive list). Some of these tools represent early attempts made to tackle the toughest challenges of metagenomic assemblies, some are abandoned and not updated anymore. Our aim was neither to show all possible tools nor benchmark them, as this is outside the scope of this review. Instead, we showcased a few widely-used and popular tools that are in constant use in the metagenomic projects.

The overall metagenomic assembly pipeline with any metagenomic assembler is quite similar to those used with ordinary assembly projects.

Typically the following four steps need to be performed:

1. QC check
2. Read trimming, adapter removal, and further filtering
3. Metagenomic assembly
4. (Optionally) Binning and analysis of the results

### QC Check

Since metagenomic assemblers cannot rely on uniform read coverage to perform coverage-based filtering of erroneous data, they end up being very sensitive to the presence of adapters, other technical sequences, and contaminations. Usually much more sophisticated algorithms are needed. This, however, means that if contamination is present, it could significantly increase both the run time and the amount of memory being used by the assembler. There are many tools that could be used to perform the initial QC check, but the most commonly used one is FastQC (Andrews et al., 2010) with further refinement via MultiQC (Ewels et al., 2016).

### Read Trimming, Adapter Removal, and Further Filtering

When working with ordinary assembly projects, the expectation is that all technical sequences and adapters should have already been removed from the input read files during the base-calling process. This, however, does not always end up being the case, since these procedures normally check for exact sequences.

Various sequencing errors and adapter read-throughs (Andrews et al., 2010) can complicate the built-in trimming procedure and, therefore, require an external trimming of adapters. There are many proven tools that could be used in these situations. The biggest thing to note is that the trimming tool needs to always be paired-end aware, which means capable of maintaining the correspondence between the left and right read files even if one read from the pair was to be completely discarded. Oftentimes, these problems end up being completely overlooked and their discovery is not made until very late during the assembly process (for example, when an assembler fails to infer the insert size distribution). Once discovered, however, the conclusion usually is that the entire assembly process needs to be restarted from scratch. Among the paired-end aware read trimming tools, we would like to highlight Trimmomatic (Bolger et al., 2014), FASTP (Chen et al., 2018), and BBDuk from BBTools suite (Bushnell et al., 2017).

Whether or not further quality trimming should be made, always remains up for debate. Each situation is different and in order to make this decision, one must understand the outcome of this procedure. Under most circumstances, light quality trimming should suffice (e.g., trim the ends of reads until, say, Q10). More stringent trimming and filtering has the potential to fragment the assemblies of species with very low abundance. On the other hand, read quality filtering of up to Q20 and even Q30 may significantly improve the run time and memory consumption of an assembler, because the majority of low-coverage data that was generated as a result of sequencing errors would be removed. All mentioned tools could be used to perform quality filtering and trimming in different modes (both tools allow specifying quality thresholds, specifying trimming and filtering parameters, and combining these settings on as needed basis).

Coverage filtering could be used to reduce the size of high complexity metagenomic datasets, by filtering out reads that likely originate from areas where the average coverage falls below a certain threshold. The resulting set of reads will most likely represent only the mostly abundant species, which also means that they will also end up being the ones assembled. Since metagenomes of high complexity tend to contain many low-abundant species, such an approach could tremendously reduce the total size of a metagenome. Various tools from the khmer software package (Crusoe et al., 2015) could be used to perform coverage filtering of input reads. The SPAdes suite of tools also contains a standalone coverage filtering tool that could be used as well.

Finally, besides quality and coverage filtering one may be interested in screening the reads for possible contaminations. Sources of contamination can be very project-specific and could include among many others human DNA, viral DNA (even PhiX from the control lane of the sequencer), and so on. Removal of contaminated reads not only improves the run time of an assembly due to a reduction in its input complexity, but could also reduce the possible number of misassemblies and other sequencing artifacts. BBDuk could be used for k-mer based filtering and masking.

### Metagenomic Assemblies With metaSPAdes

metaSPAdes was designed to address various challenges of metagenomic assembly and combines a number of new and proven algorithmic ideas from the SPAdes toolkit. The use of the toolkit is quite straightforward and we just draw attention to the few important aspects. Detailed information about metaSPAdes command line options, output, and troubleshooting guidelines could be found in Prjibelski et al. (2020).

SPAdes assembler in general and metaSPAdes in particular takes input reads via the “input libraries” abstraction. This notion should not be confused with an ordinary sequencing library, despite the fact that the terms seem to be very close in nature. The former consists of a set of reads that has a closer insert size distribution. This means that reads from the same input library could be obtained via combining several sequencing libraries provided that they are “similar” from the assembler standpoint (mean insert size and its variation, sequencing technology and error rate, etc.).

Sequencing libraries used by SPAdes as input libraries can be split into primary and auxiliary. The former are used for the construction of de Bruijn graph and further graph simplification, while the latter are used for the gap closure and repeat resolution process (e.g., long error-prone reads that are used for hybrid assemblies).

Due to the way in which the metaSPAdes algorithms work (see Nurk et al., 2017 for more information), metaSPAdes supports the usage of only one primary library that should be paired-end. Any number of auxiliary libraries (typically long noisy-reads) could be specified. metaSPAdes will use the long-range information from these libraries during the repeat resolution and gap closure steps via the dedicated *hybrid* assembly mode (see Antipov et al., 2016b for more information) to improve the contiguity of the results.

metaSPAdes allows to specify not only the locations of input data and the output directory, but also the hard memory limit (-m option) and maximum number of computational threads (-t option) to be used during the assembly. The -m option specifies the overall memory limit that metaSPAdes will never exceed. It is worth mentioning that the memory limit does not impose any limits on the internal metaSPAdes algorithms, which means that the metaSPAdes job will be killed should more RAM than had been allotted be required.

In general, it is not possible to determine just how much RAM would be required for the particular assembly job (it does not directly correlate to the size of input read files). There are many factors that could contribute to the amount of RAM being used by the process including, but not limited to: coverage read depth, the complexity of the metagenome, amount of sequencing errors/artifacts, the repeat content, the strain content, etc. The majority of these factors, unfortunately, cannot be estimated in advance.

If the metaSPAdes job has been terminated for any reason, it can be resumed via the --continue option. This option restarts the failed job from the last checkpoint using exactly the same set of options as was used before it failed. If the options need to be adjusted (e.g., change memory limit, or reduce the number of computation threads), the --restart-from option should be utilized. It allows restarting the job from the fine-grained location within the SPAdes and metaSPAdes pipeline, changing some of the options on-fly. Crucial options such as the set of input files being used will remain locked in, but it would permit adjusting options like, for example, the memory limit. In addition to this the recent version of SPAdes (as the time of writing – 3.15) supports a fine-grained checkpoint system (could be enabled via --checkpoints options) that allows to resume the work from the very last steps. Note that checkpoints increase the disk storage utilization, so one needs to use them carefully especially for large assemblies in order not to exceed the disk storage quota.

One could read about the output of metaSPAdes in detail in the metaSPAdes manual.^1^ As a quick overview, however, metaSPAdes outputs a set of consensus scaffolds and contigs (scaffolds broken at the stretches of the “N” symbol) in a standard FASTA file format. It also generates an *assembly graph* in standard FASTG and GFA formats. The assembly graph captures all of the contiguity information that is not available from the contigs. It could be further visualized using various tools including Bandage (Wick et al., 2015), SGTK (Kunyavskaya and Prjibelski, 2019), or AGB (Mikheenko and Kolmogorov, 2019).

There are few crucial steps in metaSPAdes that are important for understanding the obtained results.

#### Detecting and Masking Strain Variations

metaSPAdes collapses the majority of strain differences using a modification of SPAdes procedures for eliminating de Bruijn graph artifacts that originate from sequencing errors (in particular, the algorithms for removal of tips and bulges; Nurk et al., 2013). There are a few important differences as metaSPAdes uses more aggressive settings than the ones used in assemblies of isolates. For example, it collapses larger bulges and removes longer tips than SPAdes in normal multicell mode. Contrary to most existing assemblers, metaSPAdes does not remove the bulges; instead, it performs a *bulge projection* procedure, keeping valuable information about the collapsed bulges. This feature plays an important role for the repeat resolution approach in metaSPAdes, as will be described below.

#### Handling of Larger Strain Differences in the Assembly Graph

Unfortunately, strain differences are not only manifested as single nucleotide variations and small insertions or deletions. Often such variations are caused by highly diverged genome regions, insertions of mobile elements, rearrangements, large deletions, parallel gene transfer, etc. Therefore, the topology of the de Bruijn graph in the neighborhood of such variations is more complex than a few dozens of bulges complicating the strain variation masking procedure.

Usually, low coverage edges that are typically generated as a result of sequencing errors are removed from the assembly during the graph simplification step using a global threshold on read depth coverage. This approach, however, is not as effective when used with metagenomic assemblies because the selected threshold would need to be used not only to remove sequencing artifacts, but also the edges corresponding to rare strains, while at the same time, preserving edges corresponding to rare species. There is no global threshold that would be able to achieve this goal.

IDBA-UD (Peng et al., 2012), MEGAHIT (Li et al., 2015b), and metaSPAdes all use the coverage ratios between adjacent edges in the assembly graph to identify edges with low coverage ratios as those that most likely originate from rare strains. metaSPAdes, however, differs significantly from IDBA-UD and MEGAHIT in that instead of removing low covered rare strain edges with high-covered alternatives in the assembly graph it disconnects them to, whenever possible, preserve the information about rare strains, i.e., when it does not lead to a deterioration of the consensus backbone.

#### Utilizing Strain Differences for Repeat Resolution in metaSPAdes

**Figure 1:**
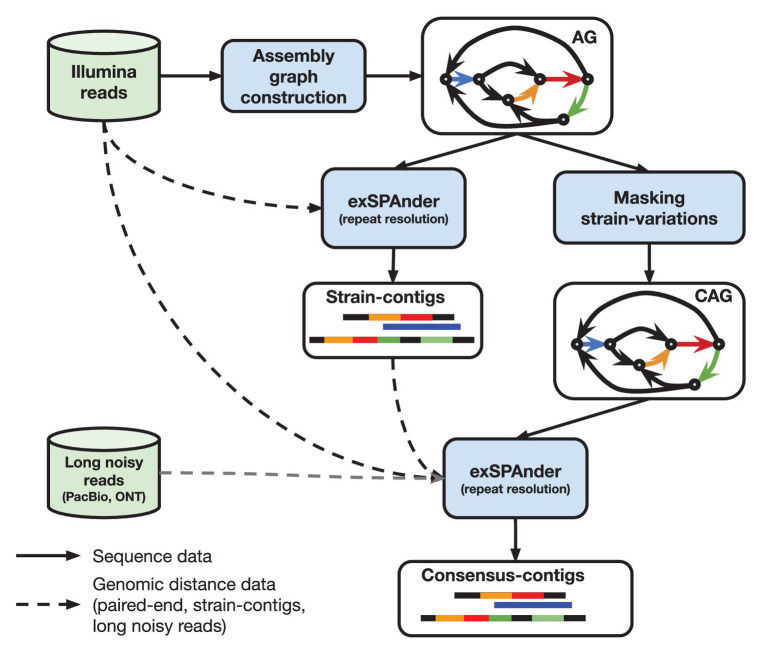
High-level overview of metaSPAdes assembly pipeline with the crucial steps and data flow outlined. “AG” denotes an ordinary (strain-level) assembly graph and “CAG” is a consensus assembly graph.

When the proof-of-concept diploid assembly mode of SPAdes was developed, it was shown that the quality of the consensus assembly of highly polymorphic diploid genomes can be improved by using the differences between the haplomes. metaSPAdes capitalizes on this idea by somewhat counter intuitively employing the differences between strains to improve consensus assemblies of strain-mixtures. In particular, contigs generated prior to masking strain-differences in an assembly graph represent genomic fragments of individual strains (strain contigs) and often provide additional long-range information for the reconstruction of a strain-mixture consensus backbone. metaSPAdes uses the following pipeline (see Figure 1; Nurk et al., 2017) that includes two launches of exSPAnder (Prjibelski et al., 2014) repeat resolution algorithm.

1. Generation of strain-contigs: Once the initial assembly graph is constructed (encoding of both abundant and rare strains), exSPAnder repeat resolution algorithm is used to generate a set of strain-contigs comprising both rare and abundant strains. The flexibility of exSPAnder algorithm allows to select settings to maximize the information about the rare strains in the strain contigs (inflating the duplication ratio of separate genomes in the assembly).
2. Transformation of the assembly graph into a consensus assembly graph: metaSPAdes generates a consensus assembly graph by identifying and masking rare strain variants.
3. Generation of strain-paths in the consensus assembly graph: metaSPAdes uses the bulge project approach that collects the information about the bulges that were collapsed during the assembly graph construction to reconstruct the paths in the consensus assembly graph corresponding to strain contigs, referred to as *strain paths*.
4. Resolution of repeats resolution using strain paths: metaSPAdes uses the modified hybridSPAdes algorithm, originally developed to work with error-prone Pacific Biosciences and Oxford Nanopore reads (Antipov et al., 2016b), to work with pseudo-reads made from the strain paths to facilitate resolution of repeats in the consensus assembly graph. Essentially, metaSPAdes performs a kind of hybrid assembly using strain-contigs.

The flexibility of metaSPAdes extends beyond the usage of strain paths alone. It can also utilize long error prone reads to generate hybrid metagenomic assemblies.

### Metagenomic Assemblies With MEGAHIT

MEGAHIT (Li et al., 2015b, 2016) was probably the first production-ready metagenome assembler that could assemble genome sequences from complex metagenomic datasets (e.g., soil) in a time- and memory-efficient manner on a single server. The success of MEGAHIT is explained by the utilization of the specialized data structure, a *succinct de Bruijn Graph* (Bowe et al., 2012), that could be used to efficiently encode a de Bruijn graph in the amount of memory closer to the theoretical minimum limit while still allowing fast graph traversal and modification.

MEGAHIT supports multiple input paired-end and single-end libraries, though it treats them identically. Also, it does not support any hybrid assemblies, essentially relying on the single kind of input.

The command line of a typical MEGAHIT run is similar to that of SPAdes: basically -1/-2 arguments could be used to provide the set of left and right paired-end reads, --12 could be used for interleaved ones and -r for single-end. While the first versions of MEGAHIT also supported GPU-based hardware acceleration, it seems that support was removed in the latest version of the assembler. The CPU version of MEGAHIT could utilize multiple computation threads that could be specified via -t/--num-cpu-threads option. The total amount of memory available to the assembly process could be specified via -m/--memory option and by default MEGAHIT uses 90% of the total memory available on the server.

The complexity, size, and contents of metagenomic datasets could be very different. By default, MEGAHIT removes all input k-mers having coverage 1 (though, some of the removed k-mers are rescued via the *mercy k-mers* approach; Li et al., 2015b), this might lead to the fragmented assemblies of low-abundant species of a metagenome. One solution to overcome this problem is to use the dedicated “meta-sensitive” preset. It could be specified via --preset option. On the other hand, large and complex metagenomes, like soil, might require lots of computational resources in order to be assembled. One way to overcome this complication is the use of a “meta-large” preset that trades the accuracy and overall recovery of species for faster computational times.

The output directory could be specified via -o/--out-dir option. The same directory keeps the intermediate results and checkpoints. So, a terminated assembly job could be restarted via --continue option. By default (and contrary to other assemblers) MEGAHIT only emits contigs having length of 200 bp and more. Should shorter contigs be required, this could be specified via --min-contig-length option. The output directory contains the set of contigs in the final.contigs.fa file.

The assembly could be visualized via the assembly graph that could be produced from the contigs via the contig2fastg option of megahit_core executable. The resulting assembly graph will be in FASTG format which is supported by, e.g., Bandage tool. If an assembly graph in more standard GFA format is desired, then additional conversion by third-party tools (like fastg2gfa from gfatools package) is required. One should note that the set of final contigs is filtered by length, so the assembly graph could be incomplete. Therefore, the intermediate contigs located in the output directory should be used for conversion instead.

### Assembling Metagenomes From Long Noisy Reads

The advent of third generation sequencing platforms provided a solution to resolve ambiguous repetitive regions in the assemblies and, therefore, significantly improve their contiguity. Despite the considerable base error rate associated with these technologies (~5–15%), their ability to produce long reads (up to 10–12 kb of median read length and more) has enabled genome assemblies with a high degree of completeness.

Typically, the assemblers that could utilize long noisy reads are based on the overlap-layout-consensus approach and, therefore, their applicability to metagenomic data with its uneven coverage, strain, and species variation was very limited. Large error rate of long noisy reads complicated the assembly problem even more (and certainly, in case of assembly of metagenomic data, no stringent coverage-based conditions that are typically employed could be used). Only very recently few tools that could be used for assembling metagenomic data have emerged. These tools include metaFlye (Kolmogorov et al., 2019), Raven (Vaser and Šikić, 2020), and latest versions of Canu (Koren et al., 2017).

The recent study of (Latorre-Pérez et al., 2020) has shown that these tools produced highly contiguous assemblies (and sometimes even complete genomes) directly from the long noisy metagenomic reads. Despite the intrinsic high error rate of these reads, final assemblies reached high accuracy (~99.5–99.8% of consensus accuracy).

We must explicitly note that the consensus quality of the assemblies obtained from long noisy reads depends a lot on the coverage of metagenomic data. And while the accuracy of assemblies of highly abundant strains could be quite good, the error rate of contigs belonging to the low-covered species could be prohibitively high.

Even more, one needs to take into account that contrary to the Illumina data, the majority of errors in long noisy reads (regardless of the technology: both PacBio and Oxford Nanopore suffer from these) are indels. Uncorrected indel errors cause frameshifts and, therefore, lead to incomplete gene prediction. Therefore, while highly contiguous assemblies produced by long read technologies indeed could be used for strain composition characterization, their applicability to further fine-grained applications such as gene prediction could be limited. For example, Latorre-Pérez et al. (2020) have shown that indel errors had a severe impact on the prediction of biosynthetic gene clusters and further polishing of assemblies are necessary. Input for polishing nanopore-based assemblies can be raw reads for Racon (Vaser et al., 2017) or Medaka,^2^ raw electric signal for Nanopolish (Loman et al., 2015), or even high quality short reads for Racon or Pilon (Walker et al., 2014). PacBio assemblies are typically self-polished via Quiver or Arrow tools or using short reads via Pilon.

To summarize: The use of long noisy reads could significantly improve the results of a metagenomic assembly often producing complete or near-complete genomes. However, the indel errors of long reads technologies hinder protein prediction (Watson and Warr, 2019) and, therefore, require further curation. We expect that the newest PacBio Hi-Fi technology with its reduced base-level error rate of >99% (Lang et al., 2020) will revolutionize the metagenomic assembly field. For now, it might be advised to use frameshift-aware tools for downstream analysis. We outline Diamond (Buchfink et al., 2015) that has frameshift-aware mode for protein prediction as well as Pathracer (Shlemov and Korobeynikov, 2019) that could align amino acid HMMs to nucleotide sequences and assembly graphs taking possible frameshifts into account.

### Binning and Analysis of Results

There are several ways in which the outcome of a metagenomic assembly could be analyzed. Most frequently, the goal is to determine the species composition of the metagenome. There are multiple tools that could be used to either perform binning or just analyzing the contents. For binning, among others, we can mention MetaBAT2 (Kang et al., 2015), CONCOCT (Alneberg et al., 2014), and MaxBin (Wu et al., 2016). See Meyer et al. (2018) for an extensive overview of various binners and binning strategies. The taxonomic profile of the assembly could be made via Kraken (Wood and Salzberg, 2014), Kaiju (Menzel et al., 2016), or Centrifuge (Kim et al., 2016). Different bins could be further refined via Binning_refiner (Song and Thomas, 2017), the results from different binning algorithms could be integrated via DAS_Tool (Sieber et al., 2018), and the completeness and possible contamination of the bins could be checked via CheckM (Parks et al., 2015). Basic assembly metrics could be obtained from MetaQUAST (Mikheenko et al., 2016). Recently several integrated pipelines have emerged that include all the aforementioned steps and combine them into a single push-button workflow. We outline MetAMOS (Treangen et al., 2013), MetaWrap (Uritskiy et al., 2018), SqueezeMeta (Tamames and Puente-Sánchez, 2019), Sunbeam (Clarke et al., 2019), among the others.

## Applications

For the purpose of this review, we are going to define several terms.

When it comes to the composition of the community of microorganisms living in certain natural conditions, we will talk about microbio**T**a. If we are focusing instead on the aggregate genome of this community, then we will be talking about the microbio**M**e. Metagenomics is a set of laboratory and bioinformatics methods for the study of microbiota and its microbiome. In many cases, the terms microbiota and microbiome are used as synonyms, but we will not repeat this error.

High-throughput DNA sequencing of short variable regions in small subunit ribosomal RNA (SSU rRNA) genes, 16S in bacteria and 18S in eukaryotes, are commonly used in most microbial ecology studies as the standard phylogenetic markers to characterize microbial diversity and evolution. However, the usefulness of SSU rRNA fragments highly depends on the underlying reference databases of SSU rRNA gene sequences that are biased toward the well-characterized ecosystems and thus result in an incomplete view of the diversity present in the actual samples. In addition, analysis of only SSU rRNA genes limits the ability to discover novel diversity, especially for Archaea and Eukarya domains, which lack good universal primers (Karst et al., 2018).

Despite considerable progress, many lineages known from SSU rRNA surveys still lack genomic representation (phylogenetic groups without cultivated representatives). Reconstruction of the genomes of individual microorganisms, and especially that of the aggregate genome of microbiota (microbiome) is a complex task, not only due to the huge amounts of data, but also to the complexity of the individual genomes. One of the main challenges at hand is the need to assemble DNA fragments of previously unknown organisms, for which there is no perfect computational solution.

Even though currently existing assembly tools are able to provide only approximate solutions, several significant discoveries have been made. Some of them greatly expanded the microbial tree of life, while others have led to more accurate clinical diagnostics and personalized treatments, the discovery of new antibiotics, improvements in oil quality, etc.

Below, we provide several examples supporting the idea that metagenomic assembly represents a more appropriate approach for uncovering new unculturable microorganisms by providing a near-complete or even complete reconstruction of their genomes as compared to the SSU rRNA analysis, which is able to answer only the question of “who is there.”

### Populating the Tree of Life With New Discoveries

The Tree of life is a visual representation of the ancestral relationships between all living organisms. The leaves of the tree correspond to living organisms, while branches demonstrate how these organisms are related to one another. Branches that are located close to each other contain closely related organisms. The Tree helps to understand the main principles behind the evolution of biodiversity: how quickly species diversify and go extinct, and what factors affect this speed. The organization of our knowledge about living things into a single evolutionary tree creates a tool that helps visualize the underlying relationships between the different species and the rules that drive the evolution of life on Earth in all its diversity.

**Figure 2:**
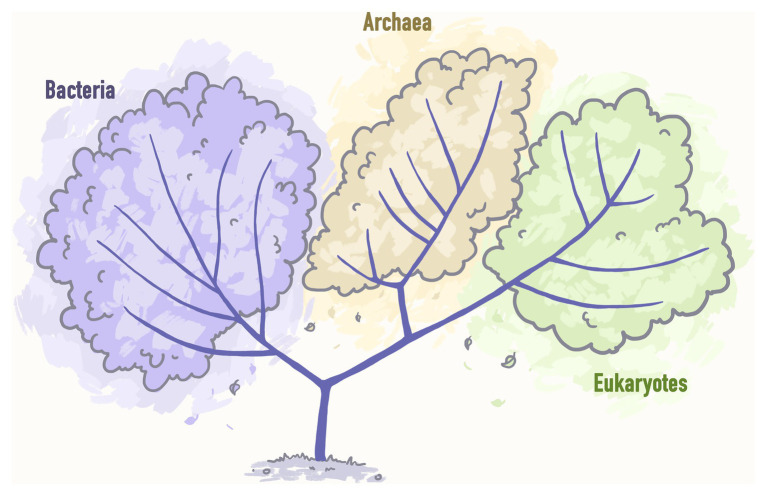
A pre-genomic tree of life representing three main domains: Bacteria, Archaea, and Eukarya. Stylized image reproduced from Woese and Fox (1997).

Woese and Fox (1997) postulated that the Tree of Life can be subdivided into three main domains: Bacteria, Archaea, and Eukarya (Figure 2).

**Figure 3:**
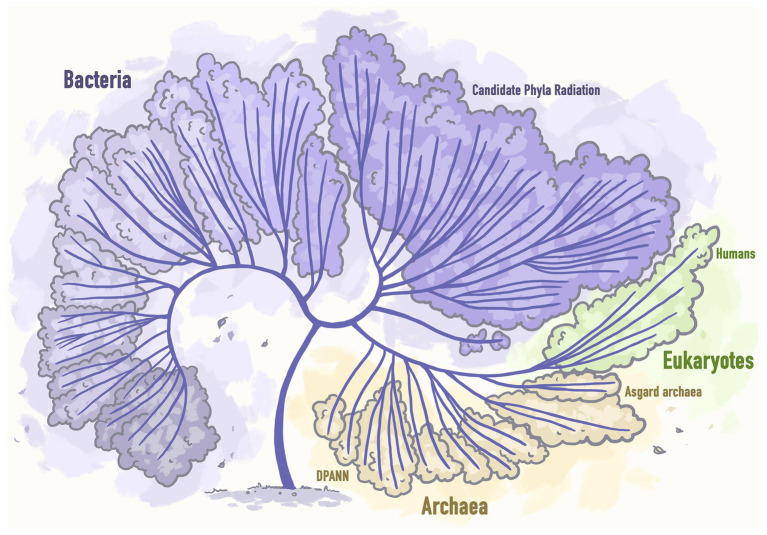
Next generation sequencing based extended tree of life representing the tree that includes 92 bacterial phyla, 26 archaeal phyla, and all 5 Eukaryotic supergroups. Stylized image reproduced from Hug et al. (2016).

Recent massive surveys of both existing metagenomic datasets created by the scientific world and available from public databases and those datasets that were specifically obtained for the purpose of conducting new studies, led to the incorporation of a good number of new genomes of unculturable microbial lineages into the Tree of life and thus its significant extension (Hug et al., 2016; Figure 3).

This incredible progress would not have been possible without the evolution of modern sequencing technologies and bioinformatics algorithms that helped to recover genomes from natural environments to predict the metabolic functions of communities of organisms that can and cannot be grown in laboratory conditions.

Thus, the massive efforts to recover the genomes of organisms from under-represented phyla allowed to discover two more microbial lineages: DPANN archaea (Castelle et al., 2015) and Asgard archaea (Zaremba-Niedzwiedzka et al., 2017). It was demonstrated that while CPR and DPANN organisms are mostly microbial symbionts, Asgard genomes encode typically eukaryotic systems. Thus, based on the phylogenetic analyses, it was surprisingly concluded that eukaryotes should be a branch within Archaea, which is a significant change to the structure of the Tree of life and of our understanding of evolution (Hug et al., 2016).

Another extension of the Tree was found in geothermal springs. Analysis of more than 5 Tb of metagenomic data of previously studied geographically distinct geothermal springs led to the discovery of a novel bacterial phylum called “Candidatum Kryptonia” (Eloe-Fadrosh et al., 2016). 4,290 metagenomic data sets from the Integrated Microbial Genomes with Microbiome samples (IMG/M) database (Chen et al., 2019) were used to determine how many microbial lineages might be missed from available sequences of existing metagenomics datasets due to mismatches with universal PCR primers traditionally used for amplifying SSU16S RNA genes to identify microbial community diversity.

A systematic survey of primer fidelity sequences recovered from over 6,000 globally sampled and assembled metagenomes containing more than 50,000 16S rRNA genes, demonstrated that approximately 10% of environmental microbial sequences are missing from classical PCR-based SSU rRNA gene surveys due to well-recognized limitations of this approach (Eloe-Fadrosh et al., 2016). Thus, there is a good chance that novel microbial phyla have so far been overlooked.

To identify novel candidate phylum, 31,955 assembled contigs longer or equal to 100 kbp that do not suffer from bias introduced during PCR amplification were selected from the above mentioned collection of 4,290 metagenomic data sets. This selection was then further reduced to 744 contigs that contained SSU rRNA gene fragments >100 bp and phylogenetically placed on a reference tree consisting of high-quality SSU rRNA sequences from bacteria and archaea (Eloe-Fadrosh et al., 2016).

The newly constructed SSU rRNA tree revealed a distinct lineage consisting of a full-length SSU rRNA sequence, while subsequent analysis of all assembled metagenomic data identified three more full-length SSU rRNA sequences.

These four bacterial genera that fell into a previously undiscovered lineage share more than 97% small subunit rRNA sequence identity on average and belong to neutral pH samples collected in Nevada’s Great Boiling Spring, the Dewar Creek Spring in Canada, and two sites in China: the Gongxiaoshe and Jinze geothermal springs. Phylogenetic analysis places this new lineage in the Fibrobacteres-Chlorobi-Bacteroidetes superfamily and suggests that this lineage represents a new bacterial candidate phylum (Rinke et al., 2013). One of the latest studies of the 1,550 publicly available metagenomes of environmental (non-human gastrointestinal) samples stored in the Sequence Read Archive (SRA) before the 31st of December 2015 allowed to produce 64,295 metagenome-assembled genomes (MAGs), of which 7,903 formed the so called Uncultivated Bacteria and Archaea (UBA) data set (Parks et al., 2017). Samples for this study were selected with the aim of focusing on the metagenomes from microbial populations with under sampled lineages.

All 7,903 genomes are estimated to be ≥50% complete and nearly half are ≥90% complete. These genomes increase the phylogenetic diversity of the bacterial and archaeal genome trees by >30% and provide the first representatives of 17 bacterial [Uncultured Bacterial Phylum 1 to 17 (UBP1–17)] and three archaeal [Uncultured Archaeal Phylum 1 to 3 (UAP 1–3)] candidate phyla. Out of all UBP and UAP, only UBP9, UAP2, and UAP3 were further taxonomically resolved using 16S rRNA gene information.

The first genomic representatives of the Terrabacteria candidate phylum SHA-109 (UBP9 genomes) were recovered from baboon feces (five genomes), palm oil effluent (one genome), a toluene degrading community (one genome), and a dechlorination bioreactor.

UAP2 phylum contains three new genomes recovered from the Tara Oceans expedition along with a single genome from the Beebe hydrothermal vent, while UAP3 is represented by a single genome recovered from a Costa Rican marine sediment metagenome and is the first representative of the ancient archaeal group (AAG) adjacent to the Lokiarchaeota.

Furthermore, metagenomic assembly helped recover 245 new genomes from the Patescibacteria superphylum (also known as CPR).

It, therefore, follows that UBA genomes significantly increased not only the phylogenetic diversity and size of the bacterial and archaeal genome trees by adding 20 new branches, but substantially expanded genomic representation within several lineages.

Recently, the ability to assemble huge amounts of metagenomic data allowed to analyze 10,450 metagenomes stored in the Genomes from Earth’s Microbiomes (GEM) catalog and sampled from diverse microbial habitats and geographic locations (Nayfach et al., 2020). As a result of this effort, a total of over 52,000 MAGs were recovered further expanding and populating the microbial tree of life by expanding the known phylogenetic diversity of bacteria and archaea.

Another study (Reysenbach et al., 2020) led to discovery of numerous never seen before life-forms living in 1,800 meters-deep volcano’s acid jets. Investigating metagenome-assembled genomes of the microbial communities inhabiting such extreme and dynamic systems 90 putative bacterial and archaeal genomic families and about 300 previously unknown genera, as well as a good number of potentially endemic to the submarine volcanic environment were identified.

Preliminary analysis (including data QC, genome assembly, and annotation and interpretation of the obtained data) demonstrated that microbial community diversity can reflect complex subsurface hydrothermal processes. In addition to updating the tree of microbial life, researchers got another tool to study Earth’s most extreme places by using the microbial community compositions as indicators of potential subsurface magmatic and hydrothermal processes.

One of the main goals of the project dedicated to the massive analysis of the human gut microbiome (Almeida et al., 2021) was to establish a comprehensive collection of microbial reference genomes and genes for accurate characterization of the taxonomic and functional composition of the intestinal microbial ecosystem as the incomplete reference data make impossible understanding of the roles of individual microbiome species and their functions and interactions. To accomplish this, authors assembled and analyzed over 200 K genomes from human gut microbiome datasets to generate the Unified Human Gastrointestinal Genome (UHGG) and Unified Human Gastrointestinal Protein (UHGP) catalogs (Almeida et al., 2021).

Different studies use different assembly approaches by creating their own state-of-the-art assembly pipeline (Nayfach et al., 2020) or using different publicly available assembly tools (Castelle et al., 2015; Zaremba-Niedzwiedzka et al., 2017; Goltsman et al., 2018; Chiu and Miller, 2019; Reysenbach et al., 2020; Almeida et al., 2021) but all of them required metagenomic assembly.

This size projects significantly contribute to numerous areas of studies providing valuable data for large-scale comparative genomics, genome, and metagenome annotation, analysis of functional potential of various environments, helping to investigate novel biosynthetic gene clusters (BGCs) encoded within the taxonomically and biogeographically diverse microbiotas.

This will help to figure out what the new microbes are and whether they have any practical applications. For example, some microorganisms could prove to be a novel source of antibiotics, have industrial applications, or maybe could be used to solve environmental problems.

As tools for obtaining genomes from various environmental samples are continually improving, we can expect that another round of studies could result in the recovery of an even larger amount of genomes.

### Plasmids and Metagenomics

Plasmids, extra chromosomal mobile elements of the microbial genomes (replicons), play an important role by providing their bacterial hosts with the ability to adapt to the environment they inhabit since they can carry antibiotic resistance genes, toxins, adhesion genes, and many others. Despite the significant role plasmids play in the environmental adaptation of microorganisms, they remain underrepresented in assembled genomic and metagenomic projects. Some plasmids remain undetected because of the incorrect gDNA isolation methods applied (Delaney et al., 2018), while many others avoid detection due to how difficult it is to assemble plasmids from genomic and especially metagenomic datasets (Antipov et al., 2016a, 2019; Rozov et al., 2017).

There is no exact definition of a plasmid from an assembly standpoint. Plasmids can be circular or linear, and can vary significantly both in size (sometimes they can be almost as large as a chromosome; Lykidis et al., 2010) and repeat content (repeats within a plasmid, repeats shared by multiple plasmids, and repeats shared between a plasmid and a chromosome). In addition, large plasmids usually present in the cell in a single copy and thus have a coverage similar to that of chromosomes, which hinders their identification as a plasmid.

Tools developed for plasmid identification in genomic data – PlasmidFinder (Carattoli et al., 2014) and cBar (Zhou and Xu, 2010) – are limited in their functionality to plasmids detection in already assembled contigs of isolates. plasmidSPAdes (Antipov et al., 2016a) and Recycler (Rozov et al., 2017) plasmid assembly tools identify plasmids as short uniformly covered cycles in the assembly graph constructed by the SPAdes assembler (Nurk et al., 2013). While both plasmidSPAdes and Recycler were able to detect a good number of novel plasmids, they have a tendency to report a significant amount of false-positives, especially in cases of non-uniform chromosome coverage. It means that the usage of these assemblers is not appropriate for metagenomic data sets, for which non-uniform chromosome coverage is typical.

Considering metagenomic data has not yet been well-studied overall, it presents a particular interest in terms of the search for new plasmids. This interest is fueled by the fact that plasmids have the ability to provide their microbial hosts, members of microbial communities, with previously unknown antibiotic resistance and various other special features necessary for the survival of the microorganisms in particular habitats. For example, more than 100 new plasmids were detected by analyzing the cecume metagenome of rats living in hospital sewage (Jørgensen et al., 2014), while during the analysis of waste metagenomes, novel plasmids providing resistance to both antibiotics and heavy metals (Li et al., 2015a) were found.

Since we believe that metagenomic datasets are a potential source of a large number of previously unknown plasmids that can be extracted from available data using bioinformatics analysis, the dedicated algorithms for plasmid assembly from metagenomic datasets are necessary. We specifically distinguish specialized assembly algorithms from the post-processing tools such as PLACNETw (Vielva et al., 2017), PlasFlow (Krawczyk et al., 2018), PlasClass (Pellow et al., 2020a), gplas (Arredondo-Alonso et al., 2020), and SCAPP (Pellow et al., 2020b) that are intended for plasmid-aware binning and classification of contigs produced by the usual metagenome assembler. One example of dedicated plasmid assembly methods from metagenomic dataset is metaplasmidSPAdes (Antipov et al., 2019), which extracts cycles from graphs of metagenome assembly (cyclocontigs) and checks whether or not they are plasmids. Since plasmids harbor a large variety of genes, the bundled plasmidVerify tool (a tool that examines the gene content of cyclocontigs and classifies them as *plasmidic* or *chromosomal* using a Naive Bayesian classifier) uses a plasmid-specific profile-HMM database to detect similarities between cyclocontigs/components detected by metaplasmidSPAdes and known plasmid-specific genes.

Using plasmidSPAdes and metaplasmidSPAdes/plasmidVerify, 22,000 bacterial genomes were assembled from the JGI GOLD database^3^ and 6,992 complete circular plasmids were obtained, 1,411 of which differ by more than 10% from the known ones (NCBI nucleotide; Antipov et al., 2016a, 2019).

### Assembling Viruses From Metagenomes

Viruses represent an integral component of the Earth microbiota and they play an important role in the health and disease of the environment they inhabit. Viruses are intracellular parasites that can live and multiply only in living cells. They infect all types of organisms, from plants and animals to bacteria. Viruses do not have their own metabolism, cellular structure, and other properties of living things. There is an opinion that viruses are an intermediate stage between living and non-living objects. In evolution, viruses are an important link in horizontal gene transfer causing genetic diversity.

Despite the fact that lots of viruses have already been identified and deeply studied, many of them remained undiscovered and several recent metagenomic studies were dedicated to improve this situation, which allowed to expand greatly the knowledge of the Earth’s virome (Paez-Espino et al., 2016; Roux et al., 2016).

However, since extracting complete sequences of viral genomes from metagenomic assemblies remains challenging, many viruses evade identification even though metagenomic datasets contain reads sampled from these viruses (Dutilh et al., 2014). Previous studies, aimed at the discovery of novel viruses, often focused on viral contigs in metagenomic assemblies and thus missed an opportunity to sequence complete viral genomes by switching from the contig-based to the assembly graph-based analysis.

From an algorithmic stand point, identifying viral genomes in metagenomic datasets is not unlike identifying plasmids since both viruses and plasmids form small subgraphs of the metagenomic assembly graphs with a very distinct topology. However, in difference from plasmids, for which multiple plasmid identification tools have been developed (see Section 3.5), there are only few specialized assemblers aimed to recover viral genomes from metagenomic datasets.

Need to mention that the task of virus assembly from a metagenome is significantly different from similar tasks for virus-only samples. The latter task has its own challenges including, e.g., highly uneven sequencing depth caused by amplification bias and huge viral population diversity.

Several assemblers including IVA (Hunt et al., 2015), PRICE (Ruby et al., 2013), and VICUNA (Yang et al., 2012) were developed for virus-only datasets. All of them could cope with the aforementioned challenges, but have their own limitations as they expect no repeats in the input data (and while it is highly unlikely to see a proper repeat in the virus genome, in the assembly repeat-like constructions could be possible due to, e.g., rearrangements and species variations) and perform various local assemblies and seed-based extensions being completely unsuitable to cope with host sequences in case of the whole metagenome.

While ordinary metagenome assemblers could be used to assemble putative virus sequences from metagenomes (Roux et al., 2017), they do not take in consideration specific genome structures of viruses that could be used by assemblers to detect putative virus sequences in an assembly graph.

Also, as we already mentioned, both viruses and plasmids form specific structures in the assembly graph, so some approaches for the plasmid extraction from a metagenome could be used for viral assembly as well.

One of the assemblers that employs an assembly graph-based approach for virus assembly from metagenomes is metaviralSPAdes (Antipov et al., 2020). metaviralSPAdes pipeline consists of three independent steps: PlasmidAssembly for finding putative viral subgraphs in a metagenomic assembly graph and generating contigs in these graphs, ViralVerify for checking whether the resulting contigs have viral origin, and ViralComplete for checking whether these contigs represent complete viral genomes. PlasmidAssembly step of metaviralSPAdes includes several important modifications to the standard metaSPAdes graph cleaning, simplification, and repeat resolution pipeline aiming to better assembly of viral sequences: additional procedures to remove strain – and subspecies – level variation, procedures to find high-coverage cycles and linear paths in the assembly graph (that would correspond to putative circular and linear viral contigs), as well as special repeat resolving mode to untangle terminal repeats that are common for linear viruses.

Another example of a specialized assembler is coronaSPAdes (Meleshko and Korobeynikov, 2020) that is aimed toward assembly of RNA viruses in general and the members of Coronaviridae family in particular from metavirome, metatranscriptome, and similar datasets. coronaSPAdes borrows different ideas from metaSPAdes, metaviralSPAdes, and rnaSPAdes (Bushmanova et al., 2019) to be able to cope with various sequencing artifacts of RNA viral datasets, as well as viral population diversity. In addition to this, coronaSPAdes employs HMM-guide genome extraction step, similar to one used in biosyntheticSPAdes (Meleshko et al., 2019) for assembly of biosynthetic gene clusters. This step uses the set of HMMs built from coronavirus proteins, aligns these HMMs to the assembly graph, and extracts the paths from the assembly graph consistent with the HMM matches and graph topology. This allows fuller reconstruction of coronavirus genomes even if multiple species are present, the rate of sequencing artifacts is high, and coverage of the genome in question is very uneven. coronaSPAdes is a leading assembler used in several high-throughput virus assembly projects including Serratus (Edgar et al., 2020).

### Miscellaneous Environments

Microbes are everywhere and no matter where we look, we will find a mixture of well-studied microorganisms that lend themselves to cultivation and completely unknown ones that do not. The thrill of possibly being first to uncover some of the Earth’s mysteries that have up to now eluded the scientific community created a boom of microbiotic studies, which in turn have begun to generate new discoveries and high-profile publications.

Iverson et al. (2012) assembled 58.5 gigabases of mate-paired next-generation metagenomic sequencing data obtained from the samples collected in the waters of Puget Sound. *De novo* assembly of metagenomic reads produced ~300 megabases of contigs that were organized in scaffolds, which in turn were binned by using tetra-nucleotide analysis into candidate genomes. These genomes were then taxonomically profiled with 16S rRNA analysis, which produced 14 candidate genomes, that included representatives of Euryarchaeota, Thaumarchaeota, Flavobacteria, and alpha-, beta-, and gamma-Proteobacteria. After a more detailed analysis, it was discovered that one of the assembled genomes represented a previously uncultured marine group Euryarchaeota of likely motile photo-heterotrophs focused on protein and lipid degradation.

Let us shift our focus and take a look at a cow. A cow’s rumen is adapted for converting plant material into energy and nutrients and this conversion is performed by enzymes encoded by the rumen’s microbiome. A recent cow rumen study (Stewart et al., 2018) analyzed more than 800 Gb of rumen metagenomic sequence data from 43 Scottish cattle that allowed to produce 913 draft assemblies of newly sequenced bacterial and archaeal strains and species. In total, all produced draft assemblies contained 69,000 proteins potentially involved in carbohydrate metabolism. More than 90% of the detected proteins were not found in public databases and thus significantly updated the pool of predicted biomass-degrading enzymes in the rumen microbiome.

This list can go on indefinitely, as every day brings new studies and new microbial discoveries, the majority of which would not have been possible without metagenomic assemblies.

## Conclusion

The science of metagenomics is the fastest growing field in modern biology. The revolution in sequencing technologies led to accumulation of huge amounts of genomic data. This, in turn, led to a clear understanding of the value of high quality reference genomes of individual microorganisms and gave the impetus to the avalanche-like growth in the number of projects related to the analysis of complex microbial communities of the environments around us.

Despite these efforts there are still no ideal laboratory methods for working with metagenomic DNA, nor a universal assembler for metagenomic data. Without a quality assembly, however, it is impossible to identify all of the members of the microbial communities being sampled, and especially those that are represented in minor amounts. Meanwhile, these yet undiscovered members play an important role not only in the life of the community itself, but also in the ways they affect the life and condition of the associated host and habitat.

Using currently available approaches, metagenomic assembly was already able to expand our overall knowledge of life and the interdependencies that define it. With continued technological advancements and increased experiences of creating more and more efficient assembly algorithms, we should expect to see an even greater number of most surprising discoveries in the years to come.

One of the most popular assemblers these days is SPAdes that is very well-set-up to accommodate future technological advancements.

## Author Contributions

AL and AK wrote the manuscript, performed the literature review and data collection, designed the figures, and revised the manuscript. All authors contributed to the article and approved the submitted version.

### Conflict of Interest

The authors declare that the research was conducted in the absence of any commercial or financial relationships that could be construed as a potential conflict of interest.

## Footnotes

^1^http://cab.spbu.ru/files/release3.15/manual.html#sec3.5

^2^https://nanoporetech.github.io/medaka/

^3^https://gold.jgi.doe.gov/
